# Virtual Care Across the Neonatal Intensive Care Continuum

**DOI:** 10.7759/cureus.35183

**Published:** 2023-02-19

**Authors:** Purnahamsi Desai, Sadaf H Kazmi, Stacey Schneider, Robert Angert

**Affiliations:** 1 Pediatrics, New York University (NYU) Grossman School of Medicine, New York, USA; 2 Child Life, New York University (NYU) Langone Health, New York, USA

**Keywords:** family-centered care, virtual care, nicu, neonatal intensive care unit, virtual medicine, telemedicine, telehealth

## Abstract

The ongoing coronavirus disease 2019 (COVID-19) pandemic has highlighted the need for establishing effective parent and family engagement throughout all aspects of medicine. Though there has been some discussion in the literature regarding the transition from typical outpatient visits to telehealth visits, there has been less written about the inpatient approach to family inclusion. Here, we seek to describe our institution’s experience with implementing virtual medicine across the full continuum of the neonatal intensive care unit (NICU) experience, including inpatient rounding, child life family visits, and outpatient high-risk developmental follow-up after discharge.

## Introduction

Telemedicine has been available in evolving form for the past 35 years. The initial use involved transmitting ultrasound images via the nascent internet to obtain a second expert opinion regarding concern for fetal anatomic anomaly [1]. Since then, many have used telemedicine visits to provide consultation to those in a rural setting far removed from tertiary care with positive outcomes and patient satisfaction [2-4]. This modality of providing care did not reach general consciousness until 2020 due to the coronavirus disease 2019 (COVID-19) pandemic. The critical requirement for social distancing prompted by the pandemic led to modifications in regulatory guidance regarding telehealth [5,6], accompanied by a reassessment of many healthcare providers of their care delivery to patients and their families [7,8]. Here, we look to describe our institution’s experience with creating and implementing virtual medicine encounters throughout the neonatal intensive care unit (NICU) course, from rounding in the NICU, to accommodating bedside virtual visits, to the Neonatal Comprehensive Care Program (NCCP), which is our high-risk developmental follow-up clinic.

## Technical report

NICU connect: virtual rounding - promoting family-centered care

Integrating parents into the decision-making process is a core principle of family-centered care [9]. It facilitates information exchange, partnering in decision-making, and fostering trust. In the pre-COVID-19 era, family presence at rounds was already inconsistent. After the onset of COVID-19, there has been a measurable negative impact on the ability of parents to be present for rounds [10]. This is due to a combination of factors, including hospital infection control policies restricting visitation, active infection in family members, and risk of asymptomatic spread of COVID-19 by a visitor. NICUs vary in their approach to balancing visitation and infection control practices, and while many restrictions remain significantly more lax than in other areas of the hospital, the unique bonds and needs of NICU parents demand that their circumstances be given extra consideration [11].

The development of video-conferencing technology on smartphones and tablet devices and its promulgation has opened up new avenues for the model of family-centered care. Virtual rounding has been studied in a pediatric intensive care unit, where its use resulted in very high overall levels of satisfaction with minimal levels of disruption. There were overall positive effects on the parent’s level of reassurance and improved communication [12]. There have been widespread reports of its use in adult intensive care units, particularly after the onset of the COVID-19 pandemic [13]. Video technology has allowed a great deal of flexibility in taking over collaborative functions; however, the technology is not risk free.

The negative consequences of introducing virtual rounding technology may include miscommunication, excessive time spent on rounds, and inadvertent disclosure of private health information (PHI). Parents may not be able to hear or see as well as if they were in person. This may make them less likely to speak up if they are unsure of what was said or have something to add. To prevent this, teams must take time to ensure proper understanding, a process that could add time to rounds above and beyond what would have been needed for in-person family-centered rounds. Due to the lack of private rooms in many NICUs, there is the possibility for the disclosure of PHI if other families are present. Families must be informed of these risks and the team must develop strategies to minimize them.

New York University Langone Health (NYULH) experience

A virtual rounding program had been planned for the NICU in the fall of 2019, just prior to the onset of COVID-19. The program plan and materials were generated by a multidisciplinary committee and reviewed by NICU family advisors and our family advisory council. The family advisory council is comprised of parents and former patients cared for at Hassenfeld Children’s Hospital at New York University (NYU) Langone and managed by Sala Institute for Child and Family Centered Care. Key elements of the program plan included providing parents information prior to the family-centered rounds (Appendix 1) to set expectations, creating educational materials for providers (Appendix 2), and creating a checklist for the team conducting family-centered rounds (Appendix 3).

**Figure 1:**
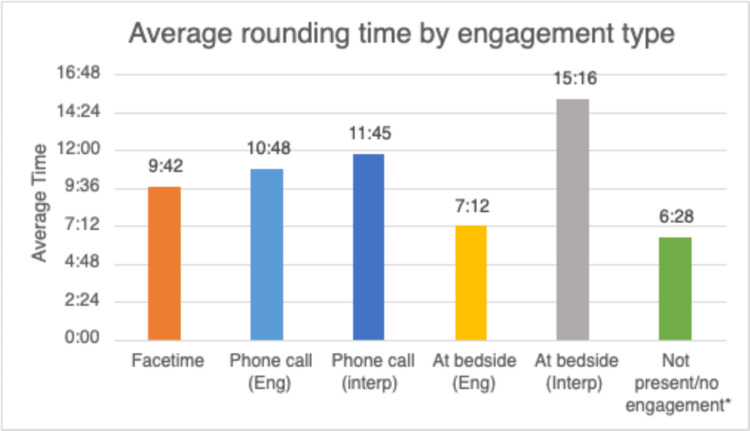
Average rounding time by engagement type Average time in minutes spent rounding per patient by engagement modality.

The technology available to the team was FaceTime via iOS on an iPad (Apple, Cupertino, California). Parents had to have an iOS device to participate in the pilot program. Concern was expressed that the virtual incorporation of parents into rounds would add excessive time to rounding. A pilot study conducted in our NICU at Hassenfeld Children's Hospital at NYU Langone from August to October 2020 indicated that on average, less than three minutes were added to each encounter (Figure 1).

Overcoming language barriers is a future challenge to be met with this technology. Attempting to use translators during virtual rounds led to prohibitive increases in time and miscommunications. In addition, the limitation of FaceTime to iOS devices is problematic. Future versions of the enterprise Electronic Medical Record, Epic (Epic Systems, Verona, Wisconsin) promise to have video conferencing capability, and future versions of iOS carry the promise of compatibility with other devices. Despite these limitations, virtual rounds allowed us to incorporate parents into rounds with unprecedented ease, adding a new dimension to family-centered care.

Virtual Visitation

We know that parents and siblings of infants cared for in the NICU require focused psychosocial support [14,15]. Child life experts are integral to that and offer support to the patient, parents, and their entire family unit, including siblings of the hospitalized infant [16]. Prior to the pandemic, we had a robust sibling support program, which included support for parents to identify the language for explaining difficult medical situations to children of all ages; pre-visit preparation and an accompanied first visit bedside with child life education about the NICU environment; therapeutic art activities to promote bonding and family togetherness; and sibling support bags for ongoing bonding from afar.

Due to the pandemic, our visitation policies were revised with restrictions on siblings and extended family members at the bedside. In an attempt to bridge this unforeseen gap, we implemented the use of technology to enhance sibling and family bonding in a variety of ways. First, we were able to continue to provide siblings the opportunity to meet the baby utilizing hospital-approved tablets and smartphones on FaceTime via iOS to offer virtual visitation. Families identified as having other children during the initial assessment are then offered further information during orientation about the opportunity for their first virtual visit bedside accompanied by a child life specialist to provide pre-visit preparation, education, and family bonding.

When a family has indicated an interest in a session, the child life specialist meets with the parents to hear about the sibling and identify any specific needs. Support is designed for each sibling based on age and developmental level, what the parent has previously shared about the new baby, questions the sibling may have, and what they will see during a virtual visit. The child life specialist then conducts a virtual visit with the sibling, educating and answering questions before seeing the baby for the first time during the virtual NICU visit. Once at the baby’s bedside, the staff helps to point out some of the medical equipment and provides clarifying information about its purpose. Conversation can then focus on engaging with the baby in a playful manner, pointing out hands, feet, eyes, hair, and other such features that help the sibling see the baby despite the medical equipment.

The healthcare team has identified some anecdotal benefits since the implementation of sibling video visits on our unit. They report that introducing siblings on video, as opposed to the use of two-dimensional pictures, has provided an opportunity for clinicians to demystify the medical environment. Additionally, siblings get to see the moving, breathing baby and how they exist in the world while asking questions and quietly speaking to their sibling for the first time. At the same time, the use of technology helps our child life specialists observe the family dynamic while also identifying fears and misconceptions. They are more informed and better equipped to make therapeutic suggestions to support the whole family throughout the infant’s stay in the NICU, similar to what had been done prior to the pandemic.

Additionally, parents have anecdotally shared that prior to having the video visits in place, siblings had asked repeatedly about the baby, not truly understanding where they were and why they were unable to come home. Seeing them virtually has provided greater context and understanding of why the baby is not yet ready to leave the NICU safely. In addition, seeing them over time appears to have the added and equally important benefit of helping the sibling to form a relationship with the baby and to feel connected, bonded, and invested in this new family member. This opportunity for virtual bonding has also been extended to grandparents and other family members with positive anecdotal feedback. One of our most important considerations when implementing the use of technology as a means of visitation in the NICU has been the benefit to parents themselves. In a few instances, we have had mothers or fathers who have been separated from the newborn due to transfer from another hospital and/or due to illness. Having virtual visits gives parents the opportunity to bond with the baby, not just to hear about the medical care but to see their child, have the baby hear their voice, and feel a part of their daily care when they cannot be at the bedside. This is mutually beneficial to the healing process and instrumental to early bonding. This also facilitated family unit bonding when our visitation policy limited family presence to one parent at the bedside at a time.

To continue to maintain safety, reduce noise, and encourage rest in the NICU environment, we have taken specific steps to identify methods to limit the possible intrusiveness of technology. We schedule visitation during specific hours to be mindful of sleep cycles, environmental noise, and patient privacy. The chosen times are typically quieter on the floor after medical rounding is complete and during care when babies are often already awake and engaged in activities. We also do so with the use of a hospital-issued device that is encrypted and measurable to ensure it maintains the appropriate approved decibel level. Finally, prior to each session, all family members are given education about stimulation to help the babies engage in a comfortable manner, limiting any undue stress that may be associated with the visit. These precautions have allowed our families a flexible approach to maintaining family unit bonding with their hospitalized infant.

Noted limitations to our virtual visitation program have included requiring advanced scheduling for an interpreter to be present at the bedside for families with limited English proficiency. The interactions take place over FaceTime without access to virtual interpreter services on the device limiting spontaneous virtual visits. Additionally, any families without an iOS device could not participate in this program, similar to our virtual rounds.

Neonatal Comprehensive Care Program: high-risk neurodevelopmental follow-up

Preterm infants are at risk for a spectrum of neurodevelopmental morbidities such as developmental delays, including language, cognitive, sensory, and motor impairment [17-19]. In addition, they are also at risk for long-term mental health issues [18] and behavioral problems, such as autism spectrum disorder [19]. It remains crucial to continue to follow this high-risk cohort of patients even during the COVID-19 pandemic, which added another layer of stress and fear for families, especially those with medically fragile children.

Our neurodevelopmental follow-up program, NCCP, follows our NYULH and Bellevue Hospital NICU graduates every six months until two years corrected age. We follow infants born <32 weeks, in addition to infants born with congenital heart disease, hypoxic-ischemic encephalopathy, and those who require extracorporeal membrane oxygenation for cardio-pulmonary failure.

Pre-COVID-19 Program Structure

Prior to the pandemic, each appointment was in-person and involved a medical check-in with a neonatologist, as well as a developmental assessment performed by a pediatric occupational therapist and a child psychologist using the Bayley Scales of Infant and Toddler Development-III (BSID-III). Recommendations were also made regarding developmentally appropriate toys and activities at each appointment.

If any concerns were noted during the medical check-in, a referral was made to the appropriate subspecialty such as pulmonology, neurology, cardiology, gastroenterology, ophthalmology, otolaryngology, or pediatric rehabilitation. Based on the developmental assessment in the clinic, referrals were also made for various therapeutic services such as physical therapy, occupational therapy, and speech and language therapy, either through early intervention or through NYULH outpatient services.

Post-COVID-19 Program Restructuring

As New York City was hit hard during the COVID-19 pandemic, it caused a shutdown of all our outpatient services for over a month, including our NCCP program. We implemented a three-phase approach to transition to seeing patients virtually to continue providing services to this cohort of patients.

Phase 1: As the NYULH system started to transition to virtual medicine and we were learning to navigate the system, we initially transitioned to telephone visits. We would call each family to discuss if they had any concerns regarding their child’s health or development and if they were receiving any therapeutic services at the time. After each phone call, we would email them resources and developmental guidelines specific to their child’s age. We documented our conversations in the medical record as a telephone encounter.

Phase 2: After a month, we transitioned to telemedicine using enterprise Electronic Medical Record, Epic. We created a process guide to be used by our physicians, occupational therapist, and child psychologist for real-time, multi-provider video visits, as well as how to document and bill. Patients were contacted in advance by the front desk staff to help familiarize them with the virtual platform. Each visit was structured in a similar fashion to our pre-COVID-19 visits. An attending neonatologist, sometimes accompanied by a neonatal fellow, would log in to do a medical check-in. Then our occupational therapist and child psychologist would log in to perform the developmental assessment. We transitioned to using the Ages and Stages Questionnaire-3 (ASQ-3) for our virtual visits as it is a reporter-based questionnaire that does not require a hands-on assessment of the child. Documentation was done as per our institution’s guidelines for telemedicine visits.

Phase 3: As transmission rates improved and outpatient services reopened for in-person visits, we transitioned to a hybrid model of seeing patients virtually and in person. Families were given the option as to which they preferred. Many families opted for virtual visits as they did not feel comfortable bringing their child to a medical office or they had left the New York City area to quarantine. All in-person appointments were appropriately spaced out to allow for social distancing and all providers were provided with appropriate personal protective equipment.

**Figure 2:**
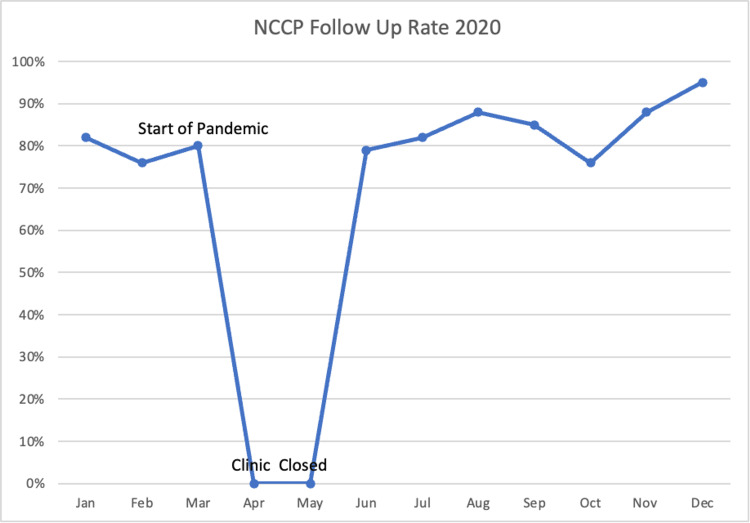
NCCP follow-up rate NCCP: Neonatal Comprehensive Care Program.

Overall, we feel that we were quite successful transitioning over to a virtual medicine platform but we did encounter some difficulties over the past year. Due to the nature of the pandemic, we lost many patients to follow-up, though rates generally returned to pre-pandemic levels following the re-opening of the clinic (Figure 2).

**Figure 3:**
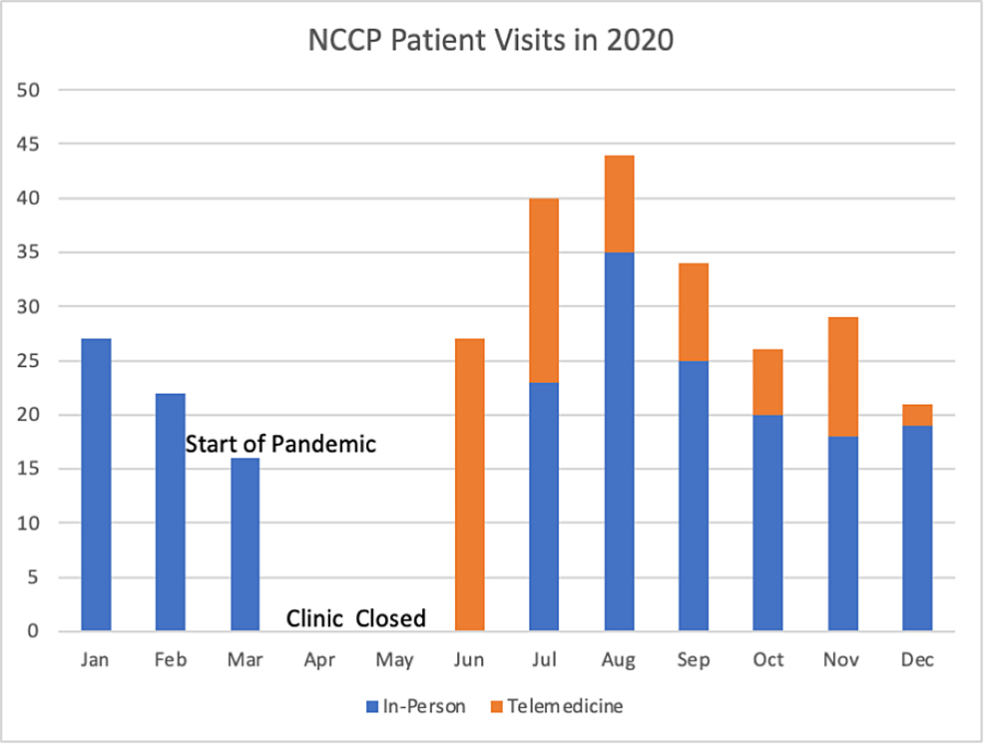
NCCP patient visits in 2020 Indicates the number of visits that occurred in 2020 (in-person versus via telemedicine). NCCP: Neonatal Comprehensive Care Program.

This was also aided in part by offering telemedicine visits, especially in the early months of the pandemic (Figure 3).

In addition, we also struggled with implementing therapeutic services for patients due to the limited number of therapists available and many patients who were already receiving services were unable to receive any therapies for months. Lastly, we know that the BSID-III remains the gold standard for developmental assessments in infants and toddlers but there was no reliable way to perform this testing virtually. Therefore, we relied on the ASQ-3 to screen for possible issues. Transitioning to a virtual medicine platform has allowed us to continue to provide important services safely to this vulnerable population.

## Discussion

Our institution’s experience with transitioning to virtual platforms across the NICU continuum proved to be successful with positive feedback from families and staff. With improvement in COVID-19 transmission rates, implementation of vaccination campaigns, and more universal masking, we have been able to liberalize our visitation policies to accommodate more in-person bonding and assessments, though many families continue to opt for virtual modalities as a means of obtaining care and participating in medical decision-making for their child.

One consideration to promote equity, compensate for historical inequities, and reduce existing disparities when designing programs for virtual services is digital access. To participate in the planned activities, there may be hidden barriers. There may be a lack of broadband availability amongst different populations. Digital literacy may be an issue for some families, and accessing a particular application or requiring a specific operating system may exclude certain populations, in particular those who have been historically discriminated against. It also takes significant resources to purchase up-to-date devices that have the capability of running the software that may be required for access. Intentional avoidance of technology might be part of a cultural difference that makes a group unable to participate in these activities. Lastly, healthcare organizations tend to choose technology that prioritizes regulatory demands, such as privacy or financial incentives that tie the technology to reimbursement [20]. These factors must be considered when choosing technologies that families will use, and soliciting feedback from family advisors or performing surveys of the patient population can help to compensate for the inequity that exists, rather than exacerbating it.

## Conclusions

Despite acknowledged limitations to universal access to and utilization of technology, virtual modalities have provided another way to more proactively partner with patients and their families in delivering family-centered care. Our experience has shown that it is feasible to implement these programs across the NICU continuum of experiences without significant detriment to workflow and with improvement in family reception of care. As we slowly move beyond the acute phase of the current pandemic and return to more in-person programming, there will still be a use for virtual care modalities given the convenience and ease that they provide. Our institution has continued to offer virtual care as described even as our visiting policies have liberalized, giving families more variety in how they choose to receive communication and participate in their infant's care. This also allows for continued flexibility in the delivery of care for providers as well. With continued use, we anticipate that further resources to optimize the technology and access to the technology will be developed, such as virtual access to interpreter services integrated into the devices used for providing virtual care.

## Figures and Tables

**Figure 4:**
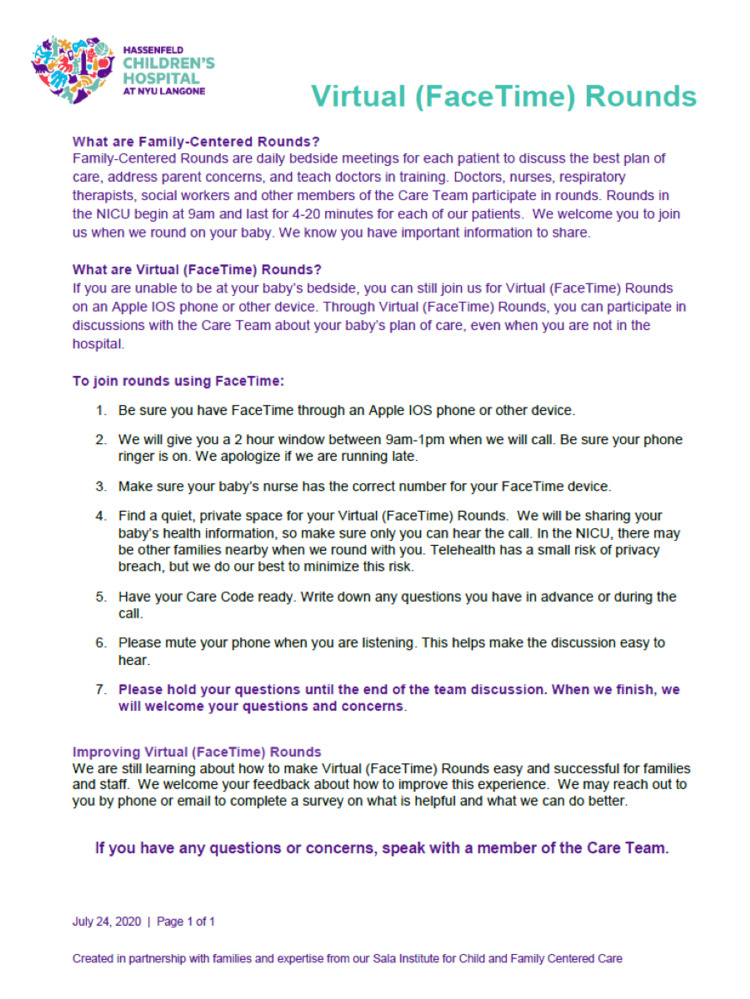
Virtual rounds parent information sheet

**Figure 5:**
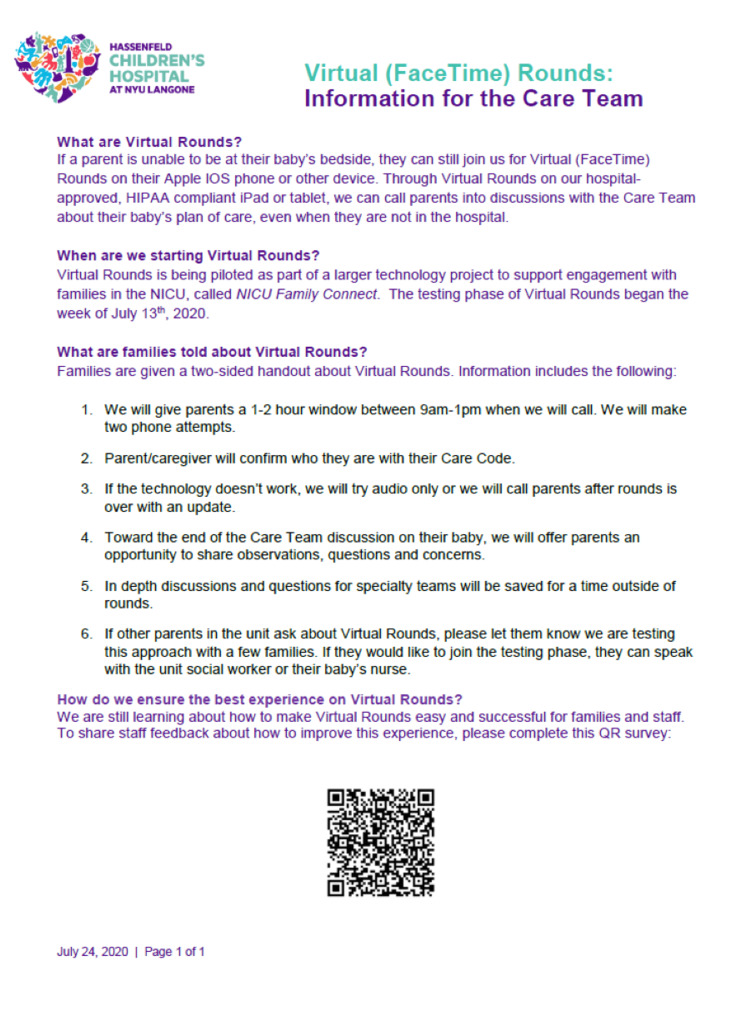
Virtual rounds care team information sheet

**Figure 6:**
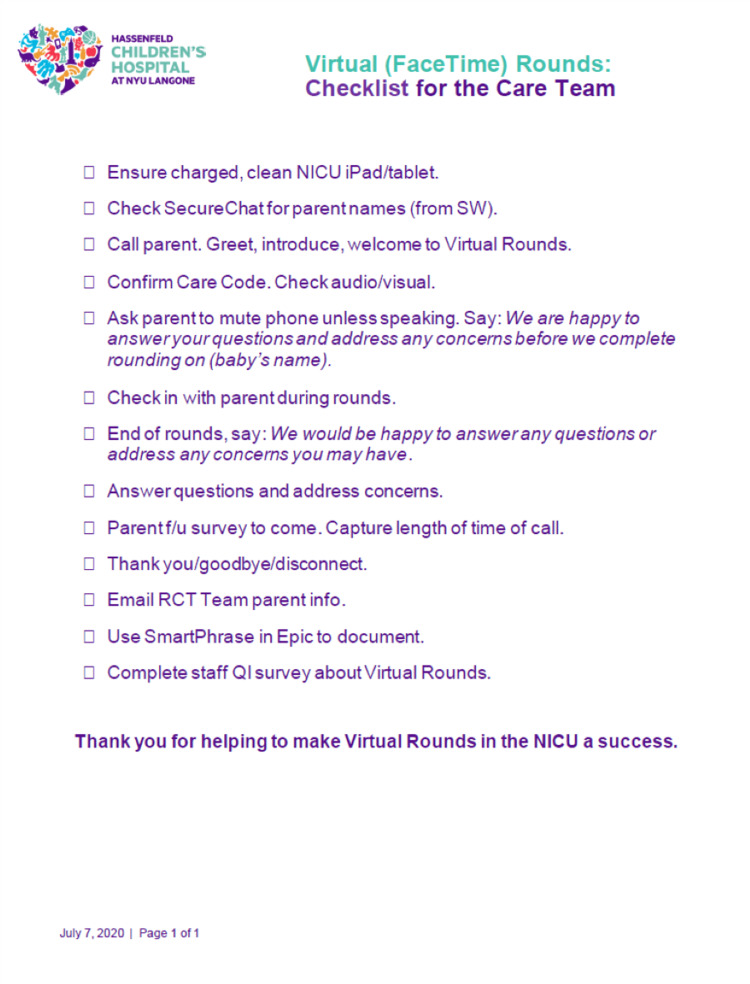
Virtual rounds care team checklist

## Appendices

Appendix 1

Appendix 2

Appendix 3
